# A Novel Hexagonal Beam Steering Electrowetting Device for Solar Energy Concentration

**DOI:** 10.3390/mi11111016

**Published:** 2020-11-19

**Authors:** Iftekhar Khan, Stefania Castelletto, Gary Rosengarten

**Affiliations:** School of Engineering, RMIT University, Melbourne, Victoria 3001, Australia; stefania.castelletto@rmit.edu.au (S.C.); gary.rosengarten@rmit.edu.au (G.R.)

**Keywords:** beam steering, solar energy concentration, electrowetting, liquid lens

## Abstract

Traditional tracking devices for solar energy applications have several disadvantages, such as bulky mechanical structure, large wind loads, and ease of misalignment. This study aims to design a flat, thin, and adaptive beam steering device to eliminate these drawbacks. A proof of concept device was fabricated to demonstrate this design. The novelty of the proof of concept device is the hexagonal structure of the electrowetting cell design. The hexagonal cell was dosed with two immiscible liquids with different refractive indices. The hypothesis of this design is that by deforming the liquid shape with the application of voltage, light can be steered and concentrated for solar energy applications. A maximum contact angle change of 44° was observed with the application of 26 V to one of the electrodes of the hexagonal cell. The device demonstrated a 4.5° change of laser beam path with only a 0.2 refractive index difference of the liquids. The 3D simulation model developed in this study shows that a tilted and flat interface can be achieved using higher dielectric constant dielectric materials. The device can facilitate the planer steering and concentration of sunlight for rooftop applications without moving mechanical parts.

## 1. Introduction

Solar energy is one of the most viable renewable energy sources on Earth. Because of the abundant availability of solar energy, this energy source for power generation is highly appealing. To harness solar energy, several devices have been developed, such as flat plate photovoltaics (PV), concentrated photovoltaics (CPV), solar heaters, etc. To capture the maximum amount of solar energy, these devices need to face the sun directly. For non-tracking flat plate collectors, the loss of solar energy can be calculated as the cosine of the angle between the incoming light and the normal to the panel. This loss of energy is defined as the cosine loss of solar energy. Reducing the cosine loss and being able to track the sun throughout the day are the primary objectives for the development of tracking systems. Tracking systems are classified into two categories- single-axis tracker and dual axis-tracker. Single-axis trackers have only one degree of freedom, which is the tracker’s single axis of rotation. Compared to a fixed-mount, a single-axis tracker can increase yearly output by 30%, and a dual-axis tracker can produce an additional increase of 10% [1,2].

Many different technologies are used to track the sun’s movement to enable the concentration of its energy and maximise yearly energy capture. The main limitations of mechanical tracking systems for solar energy collectors are cost, size, visual impact, and wind loading, particularly for applications involving mounting to buildings [3,4,5,6,7]. For example, a parabolic concentrator, along with its steering equipment, is heavy and bulky and is not suitable for rooftop applications [3,4,8]. Instead, thin and flat solar concentration devices are required for hassle-free rooftop applications. Without the need for a mechanical moving device, systems with the adaptive capability to track the sun are defined as adaptive self-tracking solar energy systems. A flat, adaptive tracking system can be easily architecturally integrated and should eliminate the need for mechanical components, large area requirements for rotation operation, and minimise installation and maintenance costs [3,4,6,7,9].

Electrowetting on dielectric (EWOD) is an emerging process with many applications, such as micro-drop generation, droplet mixing and splitting [10,11], liquid refractive index measurement [12], liquid lenses [13]. The use of electrowetting-controlled liquid lenses has emerged as a novel approach for solar tracking and concentration. By steering sunlight using thin electrowetting cell arrays, bulky mechanical equipment is not required. Electrowetting has shown to be capable of removing the drawback of mechanical solar energy tracking devices [3,4,14,15]. As such, applications of electrowetting theory for solar energy concentration and steering is a new, underdeveloped, and exciting research field.

**Figure 1:**
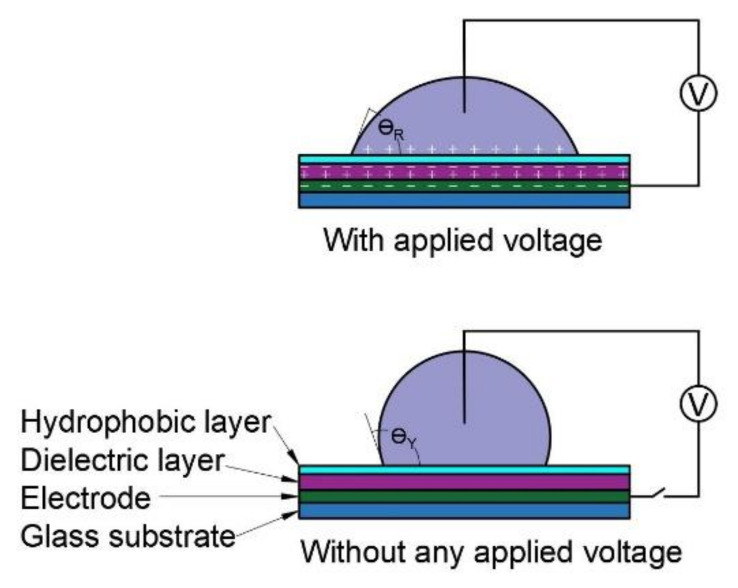
Deformation of drop by electrowetting.

Figure 1 represents a simple demonstration of the electrowetting lens and illustrates a liquid drop resting on a solid surface. The solid surface is composed of transparent hydrophobic, dielectric, and electrode layers, and a glass substrate successively from top to bottom. When the voltage is applied, the contact angle changes, and the sessile drop spreads on the surface, as shown in the figure. This change of contact angle with voltage application is known as electrowetting- on-dielectric (EWOD).

EWOD can be described using the Lippmann-Young equation (Equation (1))

(1) $$\cos\theta_{R}=\cos\theta_{Y}+\frac{\varepsilon_{0}\varepsilon_{r}V^{2}}{2d\gamma_{LV}}$$

The final contact angle $\theta_{R}$ depends on the initial contact angle, $\theta_{Y}$, the applied voltage, V, the interfacial tension between the liquid and the surrounding fluid (gas or immiscible liquid), $\gamma_{LV}$, the relative permittivity of the material, $\varepsilon_{r}$, the permittivity of free space, $\varepsilon_{0}$, and the thickness, d, of the insulating layer.

As mentioned before, recent research studies have started to provide proof of concept devices, particularly for solar energy electrowetting beam steering applications. There is still, however, a scarcity of thorough research data on electrowetting for this application. The proof of concept devices in the studies [4,14] has limitations, such as a lack of detail investigation of the liquid-liquid interface deformation. The study [16] demonstrated 360° steering of the beam using the electrowetting technique. However, the study did not investigate the design for solar energy applications. The study [17] did not investigate the liquid-liquid interface curvature with varying device parameters such as voltage, dielectric properties, etc. In most of the electrowetting device studies for beam steering applications, such as in [4,17,18,19,20], the shape of the electrowetting cell is rectangular, which can facilitate only single-axis tracking of the sun. The proposed design of [4] suggests two layers of liquid prism array directs the light to the Fresnel lens, which concentrates the light over a CPV cell. However, the study did not demonstrate any fabrication process of such structures. The study did not investigate the curvature of the liquid-liquid interface. The electrowetting solar energy tracking and concentration device of the study [20] did not identify how the individual liquid drop will be arranged in an array. Furthermore, the study did not investigate the curvature of the liquid-liquid interface.

The success of an electrowetting solar energy steering device depends on the device’s ability to deform the liquid-liquid interface. A research gap exists to investigate the liquid-liquid shape deformation in electrowetting devices for solar energy applications. Furthermore, in most previous research studies, the electrowetting cell was a square shape, which can only provide a single-axis tracking only. To harness maximum solar energy, a two-axis electrowetting device is required.

Considering the above discussion regarding the state-of-the-art beam steering electrowetting devices, the objective of this study is to design a two-axis beam steering electrowetting cell for solar energy application with the aim to investigate the liquid-liquid interface deformation by applying a varying voltage to different electrode faces. This study also aims to develop a design model to predict the liquid-liquid interface deformation with varying applied voltage, active electrode face, and dielectric constant of the dielectric material.

The design in this study hypothesizes that a thin and flat hexagonal electrowetting cell can facilitate two-axis tracking and concentration of solar energy. In the hexagonal cell, the shape of the liquid-liquid interface is changed by applying an electric potential to the electrode on the sidewall. By varying the electric potential, the liquid-liquid interface of the electrowetting cell can be altered according to the position of the sun, and thus the cosine angle of incidence loss can be eliminated. According to Snell’s law, the light changes direction when it travels through a medium of different refractive indices for not normal incident angles. Also, the difference in refractive indices of the two-immiscible liquid facilitates the change of the light path. Hence, the solar path can be tracked by using an array of thin, flat electrowetting cells. The next section discusses the advantage of hexagonal cell design and compares it with the shapes used in previous research studies.

## 2. Electrowetting Cell Shape for Two-Axis Beam Steering Application

The electrowetting cell can be designed in different shapes, such as square, circular, hexagonal, etc. In previous studies [4,14], beam steering electrowetting cells were square shapes. Wong et al. [21] compared the fill-factor of circular, square, and hexagonal cell array. The study identified that the fill-factor of the hexagonal shape array is higher than the square shape array.

**Figure 2:**
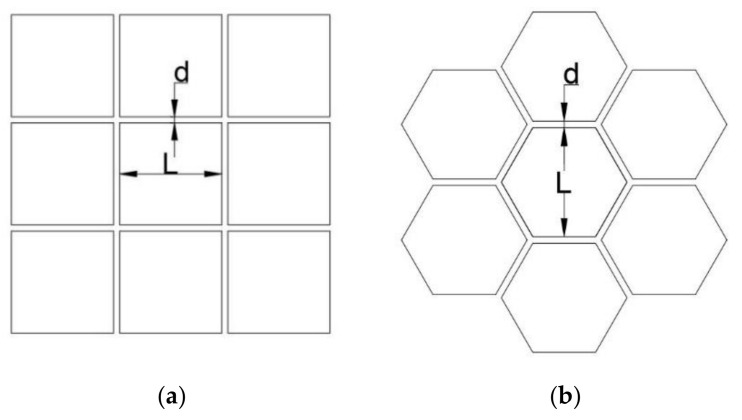
(**a**) Square cell array, (**b**) hexagonal cell array.

Most of the previous electrowetting cell array designs used square cells (such as in Refs. [4,14,22,23]). Hou et al. [22] fabricated an array of 150 µm × 150 µm square cell array, where the average wall thickness of each cell was 9 µm. To compare the fill-factor of the square and hexagonal cell array, we consider the cell array design in Figure 2.

As shown in the figure, the distance between opposite faces is L, and the thickness of the cell wall is d for both the square and hexagonal cells. Considering this, the fill-factor (*FF*) of both the square cell and the hexagonal cell is,

(2) $$FF=\frac{L^{2}}{{\left(L+d\right)}^{2}}$$

Considering the square cell size of Hou et al. [22] of 150 µm × 150 µm and an average wall thickness of 9 µm, the fill-factor of the square cell array is 0.889. Considering the same volume of a cell and wall thickness, the hexagonal cell array’s fill-factor is 0.896. Therefore, the hexagonal cell array has a slightly higher fill-factor than the square cell array. As mentioned in Hou et al. [22], by improving the deposition method, the wall thickness can be reduced to 5 µm. In that scenario, the fill-factor can be further increased.

Besides that, a hexagonal cell has the advantage of providing more degrees of freedom in steering the liquid-liquid interface in comparison to the square cell. The six faces of the hexagonal cell provide more rotational control of the liquid-liquid interface than the four faces of the square cell. In a hexagonal electrowetting cell, the liquid-liquid interface can be rotated 360° at 60° interval with the vertical axis in comparison to the 90° interval of the square shape electrowetting cell. From the above discussion, it is evident that the hexagonal cell array design has advantages compared to previous beam steering electrowetting cell array designs. Because of these reasons, this study proposes a hexagonal electrowetting cell design to ensure a higher fill factor compare to previous studies and facilitation of two-axis tracking.

## 3. Material and Fabrication Process of the Proof of Concept Device

**Figure 3:**
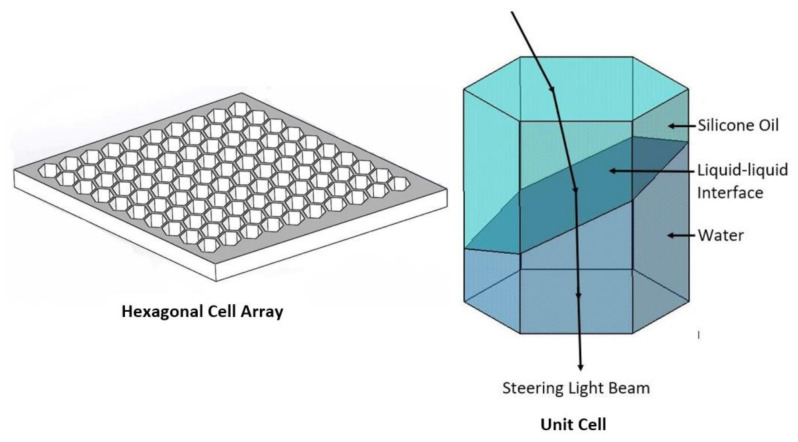
Proposed design of the electrowetting solar energy tracking and concentration device.

**Figure 4:**
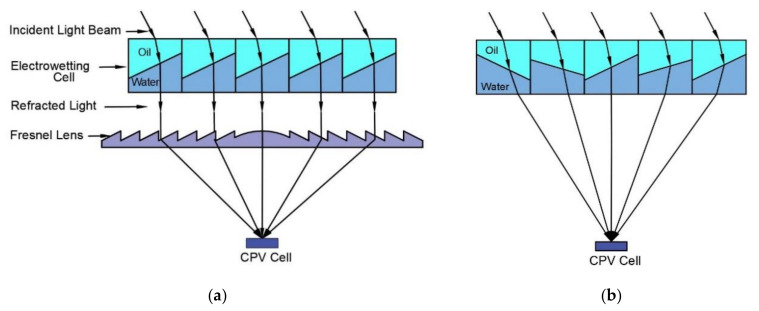
Steering and concentrating solar energy by electrowetting cell array on a CPV cell, (**a**) with a Fresnel lens, (**b**) without a Fresnel lens.

This section provides the design, material selection, and fabrication process of the proof of concept electrowetting cell unit for two-axis solar energy tracking and concentration. Figure 3 shows the proposed design of the electrowetting solar energy tracking device and the concentration system in this study. The proposed design consists of an array of hexagonal electrowetting cells that are thin (1 mm) and flat. Each cell consists of a container filled with two immiscible fluids of different refractive indices. The sidewall of each hexagonal cell is coated with separate transparent electrodes and an insulating dielectric layer. We suggest that this cell array can be installed or fabricated on top of solar energy utilization devices, such as concentrated photovoltaic cells. Figure 4 shows steering and concentrating solar energy by electrowetting cell array, (a) steering the sunlight directly to the Fresnel lens, and concentrating the light beams by Fresnel lens on a CPV cell, (b) steering and concentrating light beams on a CPV cell. As such, the system avoids bulky mechanical trackers and facilitates two-axis tracking and concentration.

The electrowetting process requires a fluid with high dielectric constant, chemical stability, and immiscibility to the surrounding medium. Furthermore, for beam steering, the fluid must have high optical transparency. Because of the high dielectric constant (80), optical transparency, and chemical stability, water is considered as the best fluid for electrowetting [3]. For beam steering electrowetting devices, the surrounding liquid to water must be immiscible, have high optical transparency, and a large refractive index difference compared to water [3]. Furthermore, it must be chemically stable. Silicone oil satisfies all these criteria [4], and thus water and silicone oil were selected as the two immiscible liquids for beam steering electrowetting device.

**Figure 5:**
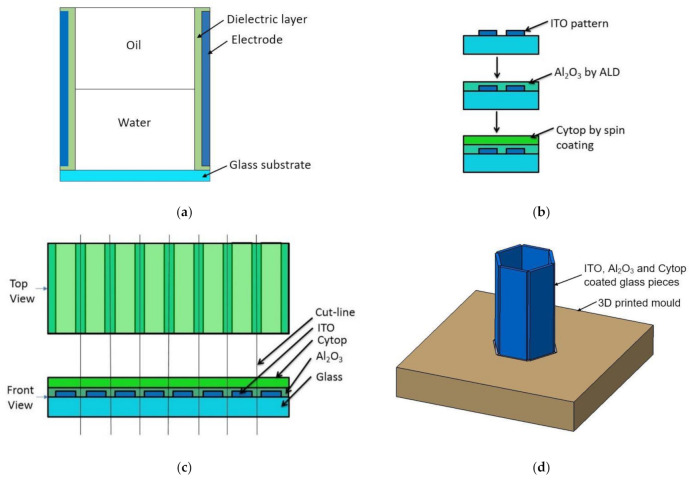
The fabrication process, (**a**) section view schematic of the cell, (**b**) layer depositon sequence, (**c**) different layers on a glass slide before dicing, and (**d**) assembled hexagonal cell of six coated glass pieces placed in a 3D-printed mould.

To deflect the liquid-liquid interface in a hexagonal cell, the interface’s triple-phase contact line must be on top of the dielectric-hydrophobic-electrode layers to achieve a change of interfacial contact angle. Because of that, the electrodes and dielectric-hydrophobic layers were deposited on the sidewalls of the hexagonal electrowetting cell (Figure 5a).

To facilitate the simple dosing of liquids and observation of the liquid-liquid interface modulation, each hexagonal cell’s face was 4.5 mm wide and 25 mm high. The long diagonal distance of the hexagonal cell was 9 mm. For this prototype, a glass slide (25 mm wide) was first coated with 4 mm wide strips of 100 nm thick Indium Tin Oxide (ITO) to form the six sidewalls of the hexagonal cell with all the layers, as shown in Figure 5b,c. The ITO electrode layer was deposited on top of the substrate (glass) using electron-beam (e-beam) deposition. To convert the ITO to a transparent layer, the sample was then annealed at 450 °C for four and a half hours in the presence of air. With this annealing, the electrode changed to a conductive layer, with approximately 80% optical transparency [24].

Recent studies [3,24] suggest that inorganic dielectric materials can improve electrowetting performance compared to organic dielectric materials, as inorganic dielectric materials have high dielectric constants and can be deposited as a thin layer. For this reason, a 100 nm thick Al_2_O_3_ layer was deposited on top of the ITO layer as a dielectric material because of its high dielectric constant and high optical transmission [24]. The Al_2_O_3_ layer was deposited by an atomic-layer-deposition process, which produced a defect-free, thin dielectric layer.

In addition, some studies [3,24,25] suggest that a two-layer dielectric-hydrophobic material can reduce defects and help to prevent dielectric breakdown. They also state that an inorganic first layer with an organic-hydrophobic second layer increases the breakdown voltage limit. In recent studies, Cytop (an organic hydrophobic material) has shown better performance in electrowetting because of its high breakdown voltage compared to other hydrophobic-dielectric materials [25,26,27]. Therefore, in this study, Cytop was chosen as the hydrophobic material to be deposited on top of the Al_2_O_3_ dielectric layer. Additionally, to improve the adhesion of Cytop to the Al_2_O_3_ layer, an adhesion promoter solution was used. This solution was prepared by mixing 0.1% amino silane agent to a mixture of ethanol (95%) and deionized (DI) water (5%). After the adhesion promoter was spin-coated, a 4% Cytop 809 solution was spin-coated and then baked to produce the 300 nm thick Cytop layer.

A 300 nm layer of Cytop was then deposited by spin coating, and the glass slide was cut along the cut lines, as illustrated in Figure 5c, to make 4.5 × 25 mm cut pieces. The cut pieces were placed in a 3D-printed mould (Figure 5d) to join the edges using an adhesive. After joining the edges, the bottom of the hexagonal cell structure was sealed using a glass slide to create a leak-free container. Each face of the hexagonal cell was 4.5 mm wide and 25 mm in height. The long diagonal distance of the hexagonal cell is 9 mm. After completing these procedures, each glass face ITO layer was connected to the external voltage supply unit using adhesive, conductive copper tape. Next, the cell was dosed with water and then silicone oil.

The next section discusses the three-dimensional simulation model developed in this study to predict the deflection of the liquid-liquid interface in the hexagonal electrowetting cell.

## 4. Three-Dimensional Simulation Model

Figure 3 shows the design concept of the electrowetting cell for solar energy beam steering applications, for which this study develops a three-dimensional model. The objective of the simulation model described below is to characterise the three-dimensional interface deflection when a voltage is applied to the hexagonal cell.

For the simulation, the Laminar Two-phase Flow Moving Mesh (LTPFMM) module of COMSOL Multiphysics software is used. The CFD module of COMSOL solves the Navier-Stokes equations to resolve the fluid motion in the electrowetting cell. The liquids in this simulation model are considered to be incompressible and Newtonian, and the flow is laminar. Also, the system is assumed to be isothermal.

With these assumptions, the mass and momentum equations for the simulations are:

(3) $$\nabla \cdot \left(u\right)=0$$

(4) $$\frac{\partial }{\partial t}(u)+\nabla \cdot \left(uu\right)=\frac{1}{\rho }\nabla p+\frac{1}{\rho }\nabla \cdot T+F_{e}+g$$

where u is the velocity vector, ρ is the density of the liquid, *p* is the pressure, **g** is the gravitational acceleration, $F_{e}$ is the external force acting on the system, and T is the total internal stress tensor, which accounts for electrothermal effects.

The Prescribed Mesh Displacement boundary condition is used in the LTPFMM physics, where mesh displacement is required to be fixed at a constant value (typically 0). The Prescribed Mesh Displacement is applied to the walls where there is no triple-phase movement (liquid-solid-liquid) contact line of the fluid-fluid interface. In the three-dimensional simulation model, the Prescribed Mesh Displacement condition is applied on the cell’s top and bottom surfaces (where dx=0, dy=0, and dz=0) to define zero displacements of the mesh in the x-, y-, and z-direction on those surfaces.

The Navier-Slip boundary condition is used on the walls in the simulation model, where the fluid-fluid interface triple-phase contact line moves due to the application of a voltage. The Navier-Slip condition allows movement of the interface along the sidewalls, and the Prescribed Mesh Displacement restricts the movement of the mesh on the other surfaces, such as the top and bottom surfaces of the cell.

In the simulation, the electric field is first calculated using the electrostatic module of the software. With the voltage assigned to the electrodes, the simulation of the electric field identifies the voltage potential at the contact line of the liquid-liquid interface. The voltage from the electric field simulation module is coupled to the LTPFMM module. The contact angle of the liquid-liquid interface with the wall of the electrowetting cell is defined by the Lippmann-Young equation. Given that water is known to have high dielectric permittivity ($\varepsilon_{r}$ = 80) in comparison to silicone oil ($\varepsilon_{r}$ = 2.2), water is the active fluid in the electrowetting cell, and thus, the water/oil interface moves and deforms. With a voltage applied, the polarised molecules of water align themselves with the applied electric field and thus exert a force on the contact line and enable contact angle modulation. The top silicone oil layer has two purposes: firstly, the higher refractive index of silicone oil (around 1.5–1.6) allows higher light beam bending angles, and second, the silicone oil functions as a protective layer for the water—to prevent evaporation.

**Figure 6:**
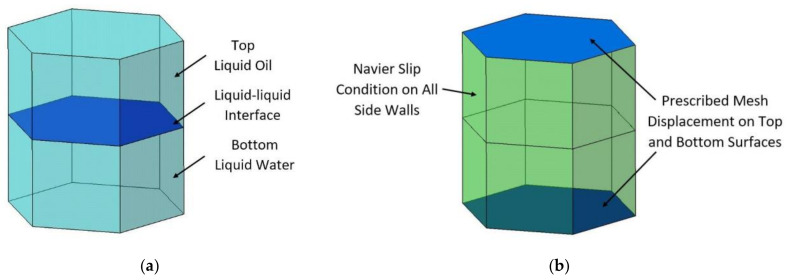
Boundary conditions of the simulation model, (**a**) liquid-liquid interface, (**b**) Navier-Slip and prescribed Mesh Displacement.

Figure 6 shows the boundary conditions of the three-dimensional simulation model. Figure 6a shows the liquid-liquid interface with water at the bottom and oil at the top. In the simulation, voltages are applied to the liquid-solid-liquid interface contact line on the vertical face of the hexagonal cell. Figure 6b shows the Navier slip condition on all side walls and prescribed mesh displacement on the top and bottom surfaces of the hexagonal cell.

## 5. Experimental Design and Results

**Figure 7:**
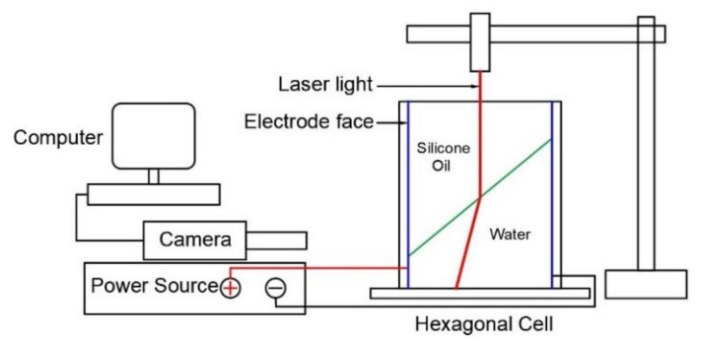
Experiment setup.

Figure 7 represents the experimental setup used in this study. There were two main experimental works conducted in this study. At first, the experiment aimed to capture images of the liquid-liquid interface’s deformation with the voltage to the hexagonal cell’s sidewall, as shown in Figure 7. As described before, the hypothesis is that with the application of voltage, the liquid-liquid interface deforms according to the electrowetting principle. In the second experimental work, the aim was to identify the deformation of the light beam. To facilitate that, we used laser light on top of the hexagonal cell, as shown in Figure 7. According to Snell’s law, this experiment hypothesizes that the light will change the path when it passes through the liquid-liquid interface of two different refractive index liquids. A camera was used to capture the change of the laser light path in this experiment. The images were then processed using image processing software on the computer.

**Figure 8:**
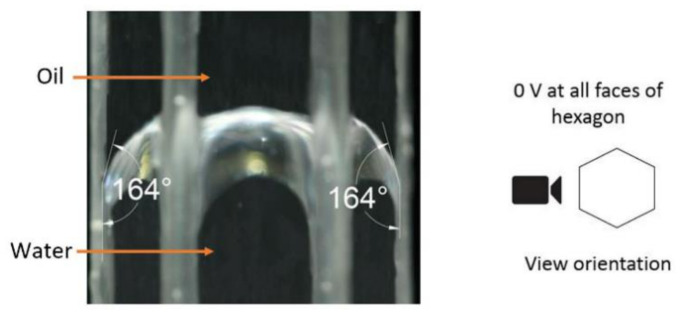
The hexagonal cell structure with an initial contact angle of 164° with 0 V on all six electrode faces.

Figure 8 shows the silicone oil-water interface without the application of external voltage, indicating the hydrophobic nature of the Cytop. The figure’s right side shows the view orientation to the cell; note that the initial contact angle was 164°.

### 5.1. Observation of Interface Modulation through Sidewalls

**Figure 9:**
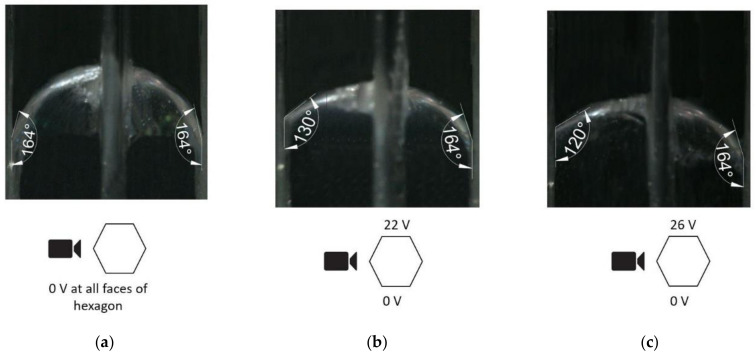
Liquid-liquid interface angle change, (**a**) 0 V, (**b**) 22 V, and (**c**) 26 V applied to the cell’s left electrode face.

After fabrication of the proof of concept electrowetting cell, we first investigated how the interface changed when the voltage was applied to only one electrode face, and the opposite electrode face was maintained at 0 V. One set of the experimental results from the repeated experiments is presented in Figure 9. As shown at the bottom of each image in Figure 9, the view was directed through the two sidewall electrode faces of the hexagonal cell, where no voltage was applied. Figure 9a shows the hexagonal cell with the initial contact angle of 164° at 0 V. As shown in Figure 9b, the contact angle changed to 130° when 22 V was applied to the left electrode face. Thus, a 34° contact angle change was observed with the application of 22 V. The applied voltage was gradually increased, and images were taken when the liquid-liquid interface was stabilized. The contact angle changed to 120° when 26 V was applied, as shown in Figure 9c. For this cell, the maximum applied voltage was 26 V, beyond which the dielectric breakdown occurred. Therefore, in this experiment, a maximum contact angle change of 44° was observed with the application of 26 V to one of the electrodes face of the hexagonal cell.

**Figure 10:**
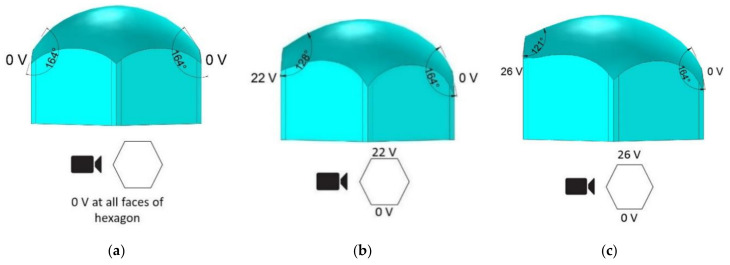
The simulation result (front view) of the bottom liquid (water) interface in a hexagonal electrowetting cell with (**a**) 0 V, (**b**) 22 V, and (**c**) 26 V applied to the cell’s left electrode face.

The experimental conditions were used as boundary conditions in the simulations. Figure 10a shows the initial shape of the water in the hexagonal cell when no voltage was applied. Figure 10b,c presents the simulation result with one electrode at 0 V with the other at 22 V and 26 V, respectively. The simulation demonstrates how the shape of the water/oil interface changes with varying applied voltages. Comparing Figure 9 and Figure 10 demonstrated that the simulation results closely align with the experimental results. Namely, for 22 V, the difference between the experimental and simulation results was only 2°, and for 26 V, the difference was only 1°.

**Figure 11:**
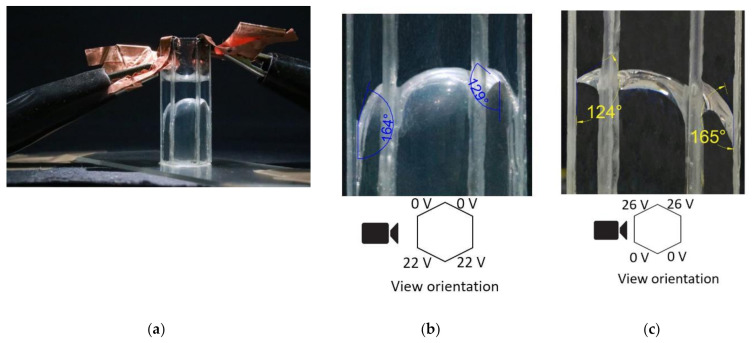
Liquid-liquid interface angle changes on the sidewalls. (**a**) experimental setup showing the contact pads used to supply voltage to the electrodes, (**b**) 22 V on the two adjacent electrode faces on the cell’s right side, (**c**) 26 V on the two adjacent electrode faces on the cell’s left side.

Next, we studied the interface changes when a voltage was applied to two adjacent electrode faces of the hexagonal cell, and the opposite electrode faces were maintained at zero voltage. One set of the experimental results from the repeated experiments is presented in Figure 11. Here, Figure 11a shows the experimental setup with the contact pads supplying the voltage to the electrodes. Figure 11b shows the interface modulation when a 22 V was applied to two adjacent electrodes faces on the right side of the cell. The contact angle changed from 164° to 129° on the cell’s right side when 22 V was applied. Figure 11c shows the interface modulation when a 26 V was applied to the two adjacent electrode faces on the cell’s left side. With this voltage, the contact angle changed from 165° to 124°.

**Figure 12:**
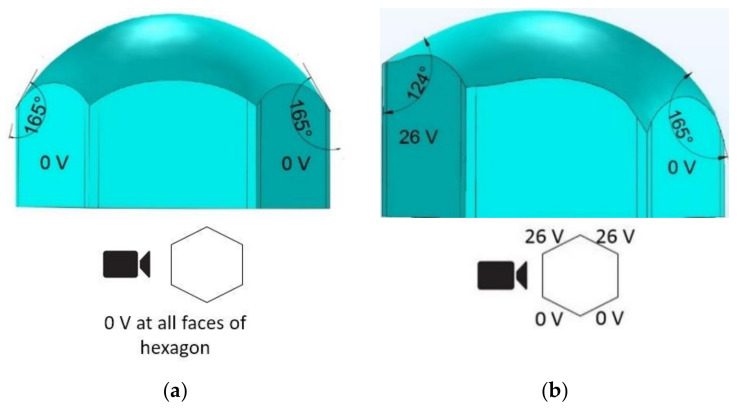
The simulation result (front view) of contact angle change of the bottom liquid (water) interface in a hexagonal electrowetting cell, (**a**) 0 V to all electrode faces, (**b**) 26 V applied to the two-adjacent electrode faces on the cell’s left side.

A simulation was conducted to compare the experimental results presented in Figure 11. Figure 12a shows the initial shape of the water in the hexagonal cell when no voltage was applied. Figure 12b presents the simulation result with applied 26 V to the two left sidewall electrode faces. As illustrated in the figure, the simulation was in exact agreement with the experimental results presented in Figure 11c.

### 5.2. Observation of Interface Modulation through Electrode Faces

In a six-face electrode hexagonal cell, the liquid-liquid interface can be modulated in different ways. Besides moving the liquid-liquid interface up and down along the sidewall, as noted in the previous section, the interface can also be rotated horizontally by applying a voltage to different electrode faces of the hexagonal cell. Thus, a two-axis interface modulation can be achieved by the hexagonal cell designed and developed in this study. The interface can be rotated 360° by changing the position of the ground and active electrodes.

**Figure 13:**
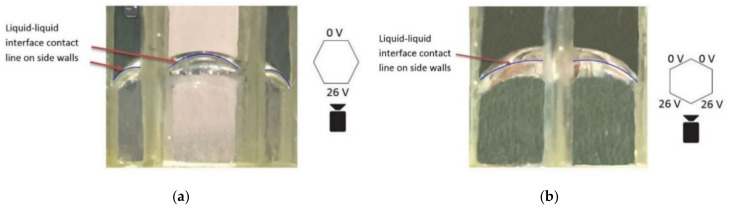
(**a**) Contact angle change viewed through the cell’s electrode face with an applied voltage of 26 V, (**b**) contact angle change viewed through the two-adjacent electrode faces with an applied voltage of 26 V.

As demonstrated in the previous sections, the interface’s contact angle change was measured by viewing the interface through the side electrode faces where no voltage was applied. View through this direction provided information on the deflection of the liquid-liquid interface on the sidewall. However, these images do not reveal the curvature of the interface. Thus, to observe the curvature of the interface, images were captured through the electrode faces of the hexagonal cell where voltages were applied. Figure 13a shows the interface shape captured through the single electrode face where 26 V was applied.

Figure 13b shows the shape of the interface captured through the two electrodes faces with an applied voltage of 26 V. Analysing these two images, it is evident that the interface was flatter with the voltage applied to two adjacent electrodes faces than the voltage was applied to a single electrode face.

These figures demonstrate that a flatter liquid-liquid interface can be formed by applying the voltage to two adjacent electrode faces, compared with the voltage applied to a single electrode face of the hexagonal cell. These findings thus reveal that for a tilted and flat interface, it is best to apply a voltage to two adjacent electrode faces of the hexagonal cell. Finally, one side of the liquid-liquid interface contact line on the sidewall can be moved vertically by varying the voltages applied to the electrode faces, and the interface can be horizontally rotated by applying a voltage to different electrode faces of the hexagonal cell. Thus, the hexagonal cell facilitates the two-axis steering of sunlight.

### 5.3. Comparison of Contact Angle Change

**Figure 14:**
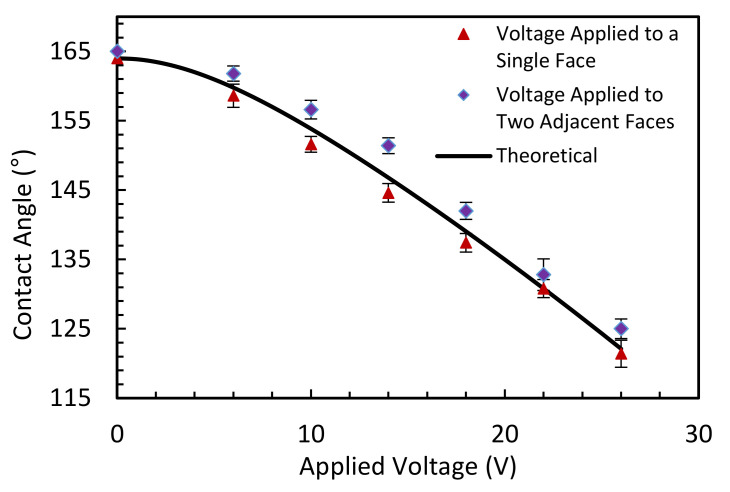
Contact angle changes of the liquid-liquid interface in a hexagonal cell with increasing voltage.

Figure 14 shows the contact angle change of the liquid-liquid interface in the hexagonal cell when the voltage is applied and gradually increased to a single electrode, and two electrode faces. Each data point in the graph represents the average of repeated five data sets, and the error bars are calculated using the standard deviation of the mean for each measurement. The theoretical contact-angle curve, according to the Lippmann-Young Equation (1), is presented in the graph. Figure 14 shows that in both the single and two electrode face experiments, the contact angle change was in close agreement with the theoretical contact angle change according to the Lippmann-Young Equation (1). The contact angle change with a single electrode face applied voltage was slightly steeper than the two electrodes faces applied voltage experiment. This may be due to the gap of ITO and dielectric layer coating between two adjacent electrode faces.

Table 1 compares the change of contact angle (Δθ = initial contact angle − final contact angle) for the theoretical equation, the simulation, and the experiment results with 22 V and 26 V voltages were applied to a single and two electrode faces. The table shows the average and standard deviation of the mean for the experimental results. As shown in the table, the simulation and experimental results were both in close agreement with the theoretical values. Hence, the simulation model can be used to predict the liquid-liquid interface modulation with varying electrowetting parameters. Furthermore, with a maximum voltage of 26 V, there was a maximum change of contact angle of 41.9° ± 1.9° in a hexagonal cell with a voltage applied to a single electrode face and 40° ± 1.4° with the voltage applied to two electrode faces.

## 6. Beam Steering

The hexagonal cell’s beam steering depends on the refractive index difference between the liquids, which should be as large as possible. Additionally, the liquid layers should be as thin as possible to reduce solar energy absorption when the light beam passes through the liquids, meaning the cell height should be as small as possible. Finally, beam steering through the electrowetting cell depends on the magnitude of the liquid-liquid interface deflection.

**Figure 15:**
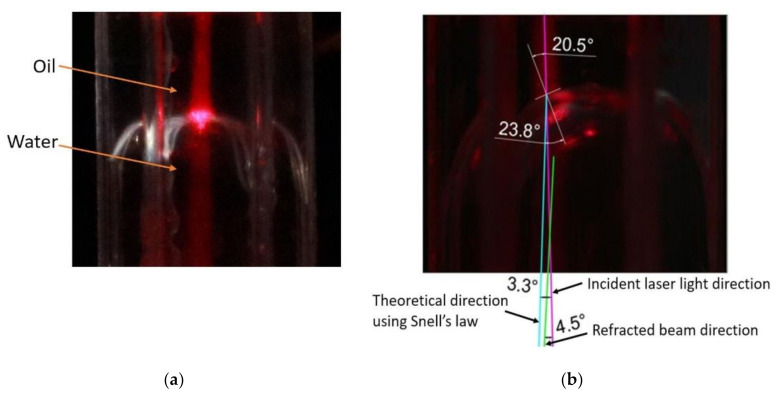
(**a**) The laser light path at 0 V and (**b**) steering of laser light due to the deflection of the interface with 26 V applied to the two right adjacent electrode faces.

The deflection of the liquid-liquid interface depends on the applied voltage, dielectric strength, and relative permittivity of the dielectric layer. As demonstrated in the previous section, when the voltage was applied to one or two electrode faces of the hexagonal cell, and the opposite electrode faces were kept at zero voltage, a tilted-curved interface was formed. With this interface, incident light will change its path and either converge or diverge depending on the refractive index of the liquids. This study used a red laser beam to investigate how the interface can change the beam’s path, as shown in Figure 15. Al_2_O_3_ nanoparticles were mixed with the water and silicone oil before dosing the cell to visualize the laser beam’s path via scattering. As illustrated in Figure 15, the laser beam first passed through the oil layer and then through the water layer. The refractive index of silicone oil was 1.5 and 1.3 for water. Figure 15a depicts the laser beam passing through the liquids when the applied voltage was 0 V. As shown in Figure 15b, the interface was deflected due to the 26 V applied to the cell’s two right electrode faces. The figure demonstrates that the laser beam had changed its path when it travelled through the interface. As illustrated in Figure 15b, there was a 4.5° change of incident laser beam path. Note, however, that the theoretical value was 3.3°. The dissolved nanoparticles in the liquids may account for the beam steering’s value being higher in the experiment than in the theoretical calculation. In this study, the liquids’ refractive index difference was only 0.2 (water and oil refractive index are 1.3 and 1.5, respectively). The light beam steering can be increased by selecting an oil with a high refractive index and by increasing the deflection of the liquid-liquid interface.

## 7. Effect of Dielectric Constant to Form Flat Tilted Liquid-Liquid Interface

**Figure 16:**
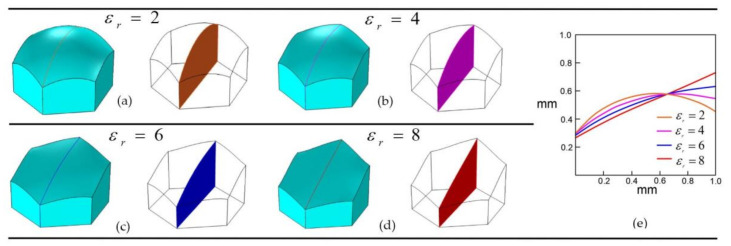
Change in the liquid-liquid interface shape and longitudinal corner plane interface with an increasing dielectric constant, (**a**) $\varepsilon_{r}=2$, (**b**) $\varepsilon_{r}=4$, (**c**) $\varepsilon_{r}=6$, (**d**) $\varepsilon_{r}=8$, and (**e**) longitudinal corner plane liquid-liquid interface profiles for different dielectric constants using the three-dimensional simulation. h.

From the Lippmann-Young Equation (1), it is evident that by increasing the dielectric constant, the deflection angle of the interface can be increased for a given applied voltage. To identify how the liquid-liquid interface behaves with an increasing dielectric constant, simulations were carried out with dielectric constant ($\varepsilon_{r}$) ranging from 2 to 8. In the simulation model, 70 V was applied to two adjacent electrodes on one side, and 40 V to two electrodes face on the opposite side of the hexagonal cell. Figure 16a–d shows the longitudinal corner plane liquid-liquid interface profiles of the three-dimensional simulation models with a varying dielectric constant. It was evident that by increasing the dielectric constant, the interface deflection increased and formed a flat and tilted surface. Figure 16e represents the simulated interface and the longitudinal section plane of the oil/water interface. With a dielectric constant of 2, the interface deflected and formed a tilted and curved shape, whereas the interface deflected as a flat surface when the dielectric constant was 8.

This parametric study of dielectric constants proves the necessity of selecting a material with a higher dielectric constant for electrowetting cells when a flat and tilted interface is required. In addition, higher dielectric constant materials reduce the need for high voltage applications to make a steeper liquid-liquid interface. The electric field concentrates more on the triple-phase (liquid-solid-liquid) contact line for materials with a higher dielectric constant. The increased electric field concentration generates an increased electromotive force, which exerts a strong outward pulling force at the liquid-liquid interface’s contact line against the inner liquid-solid interfacial tension. Because of this, a more tilted and flat liquid-liquid interface forms in an electrowetting cell for materials with a higher dielectric constant.

## 8. Conclusions

This study presents a novel hexagonal architecture and the fabrication process of a two-axis beam steering electrowetting cell. This study shows that a hexagonal electrowetting cell can facilitate two-axis beam steering via the application of voltage to the various faces of the hexagonal structure. The interface can be tilted vertically by applying a voltage to the side electrode faces. A maximum contact angle change of 44° was observed with the application of 26 V to one of the electrodes of the hexagonal cell. By sequentially applying a voltage to different electrode faces, the interface can be rotated 360° horizontally. The study also shows that it is best to apply a voltage to two adjacent electrode faces of the hexagonal cell for a tilted and flat interface. The beam steering capability of an electrowetting solar energy steering and concentration device also depends on the refractive index difference of the two immiscible liquids used in the cell. According to Snell’s law, the higher the refractive index difference between the cell liquids, the higher the beam path change. Here, in this study, the refractive index difference of two liquids (oil and water) was only 0.2. Even with this small refractive index difference, the study demonstrated a 4.5° beam steering of a red laser beam path. The three-dimensional simulation model can help predict the liquid-liquid interface modulation. It shows that a tilted and flat liquid-liquid interface can be achieved by using a high dielectric constant material for the insulating layer on the electrode. The electrowetting cell design of this study has the potential to eliminate the disadvantages associated with bulky mechanical tracking devices. The study suggests that a thin array of electrowetting cells can be placed on a Fresnel lens and direct the sunlight towards the Fresnel lens for concentration. The electrowetting cell array can also be used to steer and concentrate solar energy on a CPV cell directly.

## Figures and Tables

**Table 1:** Comparison of the change of contact angle (Δθ).

Applied Voltage	Theoretical—Using Equation (1)	A Voltage Applied to a Single Electrode Face		A Voltage Applied to Two Electrode Face	
		Simulation Result	Experiment Result	Simulation Result	Experiment Result
22 V	33.2°	36°	33.2° ± 1.3°	37°	32.2° ± 2.3°
26 V	41.9°	43°	41.9° ± 1.9°	41°	40° ± 1.4°
